# Comparisons of Visual and Surgical Outcomes after Reuse or Replacement of Dislocated in-the-Bag Intraocular Lens

**DOI:** 10.1155/2018/7342917

**Published:** 2018-03-28

**Authors:** Takayuki Baba, Tomohiro Nizawa, Toshiyuki Oshitari, Shuichi Yamamoto

**Affiliations:** Department of Ophthalmology and Visual Science, Chiba University Graduate School of Medicine, Chiba, Japan

## Abstract

**Purpose:**

To compare the visual and surgical outcomes after a reuse or a replacement of a dislocated in-the-bag intraocular lens (IOL).

**Methods:**

This was a retrospective, nonrandomized case series at a single ophthalmological institution. Cases with an in-the-bag dislocation of an IOL were treated by pars plana vitrectomy and the reuse or the replacement of the IOL. The lens was held by intrascleral fixation of the haptics of the IOL under both conditions. The same dislocated IOL was reused in 6 eyes (group A) or it was replaced with another IOL in the other 9 eyes (group B). The pre- and postoperative parameters analyzed included the visual acuity, refractive error, corneal endothelial cell density, and intraocular pressure (IOP).

**Results:**

There was no significant difference between the two groups in the postoperative visual acuity (*P* = 0.388), refractive error (*P* = 0.955), IOP (*P* = 0.529), and endothelial cell loss (*P* = 0.940). A breakage or a tilting of the IOL was observed and required replacement in three eyes in the reuse group (*P* = 0.044).

**Conclusions:**

Half of the cases with reused in-the-bag dislocated IOL had a breakage or a tilting of the IOL. The replacement of the in-the-bag dislocated IOL is better than the reuse of the IOL with intrascleral haptics fixation.

## 1. Introduction

A dislocation of an intraocular lens (IOL) is usually treated by repositioning the IOL mainly by two methods: one is the extraction of the dislocated IOL and replacement with a new IOL in the anterior or posterior chamber, and the other method is to reuse the dislocated IOL. The IOLs are fixed to the sclera by nonabsorbable sutures [1]. In the former method, an iris-claw IOL [2] or an open-loop IOL [3] is placed in the anterior or posterior chamber. These two methods have their unique complications. The anterior chamber IOL can be associated with a reduction in the corneal endothelial cell density and occasional bullous keratopathy [4]. On the other hand, the reuse of the IOL requires more complicated surgical procedures and longer surgical times, and a breakage of the sutures can occur long after the surgery [5].

Two recent studies reported on an intrascleral fixation of the haptics of the IOL with long-term stability of the IOL without using sutures [6, 7]. Even in eyes with a dislocated IOL, an intrascleral fixation of the same IOL has been reported to be effective by Kim et al. [8]. They reported that most of the cases had a dislocated out-of-the-bag IOL, and the course of eyes that underwent refixation of an in-the-bag dislocated IOL using the intrascleral fixation technique was unknown.

The purpose of this study was to compare the visual outcomes after a reuse of the in-the-bag dislocated IOL to that after replacing the dislocated IOL. In both cases, the IOL was held by the intrascleral fixation technique which we believe is simpler and safer than the conventional IOL suture technique.

## 2. Patients and Methods

This was a chart review of 15 eyes of 15 patients with a dislocated in-the-bag IOL. The cases were consecutive and treated by a single surgeon (TB) at the Chiba University Hospital from August 2015 to December 2016. Eyes with ocular trauma or with mild dislocation of the IOL that did not require surgical repair were excluded. Dislocated IOLs that were fixed at the ciliary sulcus (out-of-the-bag) were also excluded. Some cases had good corrected vision, but they wanted to undergo surgery because of severe hyperopia.

The cases were divided into two groups: in group A, the dislocated IOL was refixed using the intrascleral fixation technique, and in group B, the dislocated IOL was removed and a new IOL was inserted and fixed by the intrascleral fixation technique. If the dislocated IOL was a single-piece IOL, it was replaced with a new IOL. Otherwise, the dislocated IOL was reused. In other words, all of dislocated 3-piece IOL were planned to reuse. However, in two eyes, the dislocated IOLs were broken during surgery and replaced. These two eyes were placed in group B.

The procedures used in this study were approved by the Institutional Review Board of Chiba University Graduate School of Medicine (number 2620), and they conformed to the tenets of the Declaration of Helsinki. Patients were informed on the use of their clinical data, and they were allowed to opt out from the study at any time.

The surgical techniques were different in the two groups. In group A, a 3- or 4-port pars plana vitrectomy with 25-gauge system was performed. After core vitrectomy, the lens capsule was removed by forceps and a vitreous cutter under chandelier illumination. The residual lens cortex was removed by a vitreous cutter with reduced cutting rate of 800 cuts/min. Then two sclerotomies were made with a 25-gauge blade separated by 180 degrees at 3:30 and 9:30 o'clock. Each sclerotomy was 2 mm from the limbus. The haptics of the IOL were pulled out from the sclerotomies by 25-gauge forceps (G-S03673, Geuder, Heidelberg, Germany) and fixed in the scleral tunnels. The positions of scleral tunnels were at 4:00 and 10:00 o'clock, and the tunnels were made parallel to limbus. The scleral tunnel was made at a half-thickness of sclera to prevent a later exposure of the haptics. The length of scleral tunnels was about 5 mm, and glue was not used to fix the haptics.

In group B eyes, 3-port pars plana vitrectomy was performed with 25-gauge instruments, and the dislocated IOL was removed through a limbal incision. Because the IOL was made of acrylic or silicone, the IOL was cut in half and removed through a 3 to 4 mm sclerocorneal incision. After extracting the IOL, a new foldable 3-piece IOL was implanted and fixed by the same technique used for group A eyes. The goal diopters were set as the same diopters of fellow eyes.

The following parameters were measured at the baseline and the postoperative period: best-corrected visual acuity (BCVA), refractive error, intraocular pressure (IOP), and density of corneal endothelial cells. The visual acuity, refractive error, and IOP at the final visit were used for the statistical analyses. The size of sclerocorneal incision and the intra- and postoperative complications were also recorded. The refractive error was measured with a TONOREF II autorefractometer (Nidek, Aichi, Japan), and the IOL power was determined by an OA-1000 optical biometer (Nidek, Aichi, Japan). The density of the corneal endothelial cells was measured by an EM-3000 specular microscope (TOMEY, Aichi, Japan) at one month postoperatively.

### 2.1. Statistical Analyses

The statistical analyses were performed with the SPSS ver.20 (IBM Japan, Tokyo) software. The significance of the differences in the visual acuity, refractive errors, and IOP was determined by Wilcoxon tests. The significance of differences in the incidence of complications was tested by chi-square test. A *P* value < 0.05 was taken to be significant.

## 3. Results

The demographics of the patients are presented in Table 1. There were no significant differences between groups A and B in the age, axial length, time after the implantation of the IOL, preoperative visual acuity, and IOP. The number of cases with subluxated IOLs with opaque capsule which significantly disturbed central vision was larger in group A. There were no significant differences between the two groups in the postoperative parameters including the visual acuity (*P* = 0.388, Table 2), refractive error (*P* = 0.955), amount of astigmatism (*P* = 0.689), and IOP (*P* = 0.529). The difference between the goal diopter and the postoperative refractive error was greater in group A (5.1 ± 4.7 versus −0.5 ± 0.4), but the difference was not statistically significant (*P* = 0.607). The change of astigmatism was 1.1 ± 0.7 in group A and was 0.5 ± 0.2 in group B, and this difference was not significant (*P* = 0.364). The reduction of the corneal endothelial cell density was 1.8 ± 2.9% in group A and 3.7 ± 6.0% in group B (*P* = 0.940).

**Figure 1:**
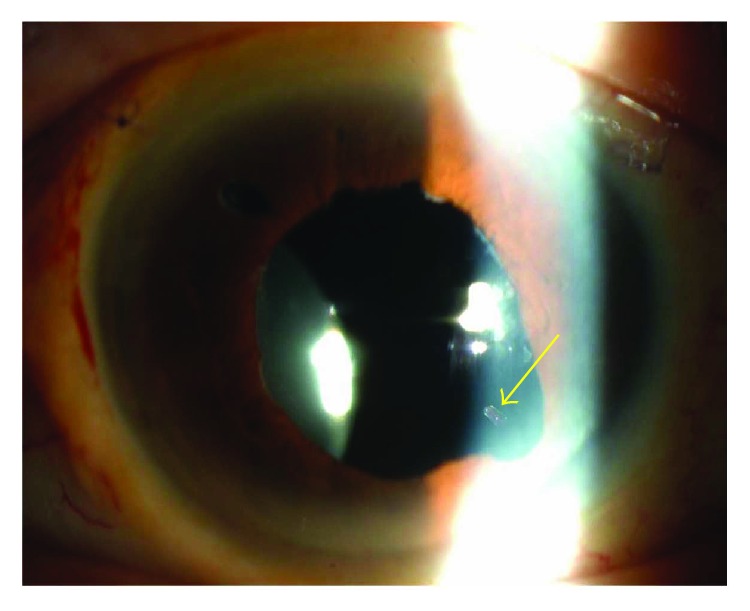
Separation between the haptic and optic in an eye with a dislocated intraocular lens. The patient was aware of the sudden decrease of vision in his left eye which had undergone repositioning of an in-the-bag dislocated IOL using the intrascleral haptics fixation technique two weeks earlier. Note that the optic has shifted nasally and the end of the haptic formerly inserted into optic can be seen (arrow).

The dislocated IOL was broken during the surgery in two eyes of group B. The dislocated IOLs had been planned to be reused in these two eyes, but the broken IOLs were removed, and new IOLs were implanted and fixed intrasclerally. Two cases in group A had a separation of the optic and the haptic at two weeks and one year after the surgery (Figure 1). These patients did not have trauma to the eye according to the patients. Another case in group A had a tilting of the IOL at one week after the surgery. The incidence of the IOL-related postoperative complications was high in group A (50%), and the difference was statistically significant (*P* = 0.044). These three cases eventually underwent surgery to replace the IOL. No other complications including vitreous hemorrhage, retinal detachment, glaucoma, and endophthalmitis were observed during the study period.

## 4. Discussion

The results showed that there was no significant difference in the visual acuity, refractive error, IOP, and the density of corneal endothelial cells in the two groups of eyes. On the other hand, the incidence of tilting and broken IOL was higher in the eyes with a reuse of the IOL.

The advantages to reusing the dislocated lens are as follows: the sclerocorneal incision is not necessary and may result in lower surgery-induced astigmatism, reduced risk of endothelial cell loss because of less manipulation in the anterior chamber, and shorter surgical times [8]. However, the postoperative astigmatism was not significantly different between the two groups. A small incision of 3 to 4 mm should not induce severe astigmatism. The endothelial cell loss was also not significantly different between the two groups. The careful manipulation of the dislocated IOL when it was removed prevented a reduction in the corneal endothelial cell density. Lastly, the surgical time was longer in group A because the removal of the capsule and remnants of the lens cortex consumed significant time. Collectively, the advantages of reusing the dislocated IOL were not significant in these cases.

We used intrascleral fixation for the in-the-bag dislocated IOL. An IOL dislocated in-the-bag has been reported to be distorted by the contracted capsule [9]. The haptics can be bent by the long period of compression by the lens capsule and never return to their original shape. This bending of the haptic may be one reason for the tilting of an IOL after intrascleral fixation. The other reason for a tilting of the IOL is the short length of the IOL. Because we did not know the model of IOL implanted in some cases, the length of the IOL might not have been long enough to be fixed by the intrascleral haptics fixation technique. The IOLs with short loops have to be pulled by force when fixed intrasclerally, and this can result in a tilting of the optics.

Two cases had a separation of the haptics and optic during the surgery, and two cases had it postoperatively. The junction between the optic and haptics can become weaker after years of implantation [10, 11]. Generally, the haptics are simply inserted into the narrow space between the optic and no adhesive material such as glue is used. The mechanical stress is applied especially when the haptics are pulled out through the small scleral incisions. Four of our cases had undergone cataract surgery and implantation of the IOL at 7, 8, 10, and 11 years before the in-the-bag dislocation. For the eyes with IOL implanted many years ago, it is necessary to be very careful in handling the haptics during surgery using the intrascleral fixation technique to prevent damage of a weakened IOL.

There are some limitations of this study. First, the number of cases was small. The cases of in-the-bag dislocation of IOL are not so common, and this study included only cases treated by a single surgeon at a single institution. These conditions can reduce the biases related to the surgical skills. However, it is difficult to present that there is clinical significance from statistical difference between groups and to draw conclusions which surgery method had the superiority for in-the bag IOL dislocation because the sample size is too small in this study. Second, this was a retrospective study based on a chart review. We used the eyes with replacement of a dislocated IOL as controls although those eyes with replacement of IOL included eyes with damaged IOL which we decided not to reuse during surgery. To confirm the results of this study, a prospective randomized study comparing eyes with or without replacement of an in-the-bag dislocated IOL using intrascleral fixation technique needs to be conducted.

In conclusion, we investigated the outcome of intrascleral haptics fixation of an in-the-bag dislocated IOL. The postoperative visual acuities and intraocular pressures were not significantly different in eyes with a replacement of the IOL. However, the incidence of postoperative damage and tilting was higher in the eyes with a reuse of the in-the-bag dislocated IOL. We recommend that surgeons replace the IOL instead of reusing it in cases with an in-the-bag dislocated IOL by intrascleral haptics fixation.

## Figures and Tables

**Table 1:** Patient demographics at the baseline.

	Group A	Group B	*P* value
Number of eyes	6	9	N/A
Age (year)	62.3 ± 17.3 (Median: 64)	67.0 ± 10.9 (Median: 67)	0.689
Axial length (mm)	24.5 ± 1.7 (Median: 25.5)	25.7 ± 2.5 (Median: 25.7)	0.388
Time from IOL implantation (year)	9.7 ± 4.4 (Median: 9)	7.3 ± 5.5 (Median: 7)	0.224
Best-corrected visual acuity (logMAR)	0.34 ± 0.34 (Median: 0.40)	0.08 ± 0.20 (Median: 0)	0.088
Refractive error (diopter)	6.2 ± 5.1 (Median: 8.4)	4.1 ± 1.7 (Median: 5.3)	0.689
Astigmatism (diopter)	0.7 ± 0.3 (Median: 0.5)	0.9 ± 0.2 (Median: 0.8)	0.388
Intraocular pressure (mmHg)	15.3 ± 4.1 (Median: 16)	16.7 ± 3.6 (Median: 17)	0.529
Observation period (month)	13.2 ± 4.8 (Median: 13)	11.8 ± 5.7 (Median: 11)	0.388

Mean ± standard deviation. logMAR: logarithm of minimum angle of resolution.

**Table 2:** Surgical outcome of the studied cases.

	Group A	Group B	*P* value
Best-corrected visual acuity (logMAR)	0.21 ± 0.34 (Median: 0.07)	0.05 ± 0.12 (Median: 0)	0.388
Refractive error (diopter)	1.8 ± 11.6 (Median: −2.0)	−2.8 ± 2.8 (Median: −1.8)	0.955
Difference between the goal diopter and postoperative refractive error (diopter)	5.1 ± 4.7 (Median: 0.3)	0.5 ± 0.4 (Median: 0.4)	0.607
Astigmatism (diopter)	1.8 ± 1.4 (Median: 1.6)	1.4 ± 0.9 (Median: 1.3)	0.689
Intraocular pressure (mmHg)	14.3 ± 3.7 (Median: 16)	13.2 ± 3.2 (Median: 14)	0.529
Reduction of corneal endothelial cells (%)	1.8 ± 2.9 (Median: 0.5)	3.7 ± 6.0 (Median: 0.4)	0.940
Incidence of postoperative complications (eye)	3	0	0.044
Breakage of IOL	2	0	
Tilting of IOL	1	0	

Mean ± standard deviation. logMAR: logarithm of minimum angle resolution.
